# TAFRO Syndrome Associated with EBV and Successful Triple Therapy Treatment: Case Report and Review of the Literature

**DOI:** 10.1155/2016/4703608

**Published:** 2016-09-29

**Authors:** Malorie Simons, Emmanuel Apor, James N. Butera, Diana O. Treaba

**Affiliations:** ^1^Department of Medicine, Rhode Island Hospital and Warren Alpert School of Medicine, Brown University, Providence, RI, USA; ^2^Department of Hematology and Oncology, Rhode Island Hospital, Providence, RI, USA; ^3^Department of Pathology, Rhode Island Hospital, Providence, RI, USA

## Abstract

TAFRO syndrome is a rare constellation of symptoms: thrombocytopenia, anasarca, reticulin fibrosis of the bone marrow, renal dysfunction, and organomegaly. Its pathogenesis involves an excessive and inappropriate cytokine storm, most notably from IL-6, causing multiorgan failure; however, its etiology is undetermined. Starting in 2012, TAFRO syndrome was first identified in Japan as an atypical variant of Castleman's disease. Previous reports include various different treatment protocols with inconsistent survival outcomes. Here we report the first known American, EBV positive but HIV and HHV-8 negative, male with TAFRO syndrome. He was successfully treated with an unusual three-drug regimen including tocilizumab, etoposide, and rituximab. We review the literature of TAFRO syndrome, discuss its possible viral etiology, and propose an original treatment regimen.

## 1. Introduction

An extremely rare constellation of symptoms, the TAFRO syndrome includes thrombocytopenia, anasarca, reticulin fibrosis of the bone marrow, renal dysfunction, and organomegaly. In 2012, TAFRO syndrome was first identified in Japan as an atypical variant of multicentric Castleman disease (MCD), a group of disorders associated with systemic inflammatory symptoms, reactive proliferation of benign lymphocytes, and multiple organ impairment secondary to proinflammatory cytokines [1]. To date, there have been up to 25 reports of TAFRO syndrome, most of which were in Japan, with different treatment protocols and survival outcomes.

The etiology of TAFRO syndrome is unclear, although it is reasonable to propose that there is an insult to the immune system that produces hypercytokinemia. Human herpes virus-8 (HHV-8) is a known cause of this cytokine storm in HIV positive MCD patients and some HIV negative patients [2]. In addition, Epstein-Barr virus has also been associated with Castleman disease [3, 4]. However, to date, there is no known cause of TAFRO syndrome.

Due to the rarity of TAFRO syndrome, there are no set guidelines on treatment although leading experts are beginning a multicenter retrospective clinical study to start this process [5]. Previous treatment efforts have centered on anticytokine therapy, namely, Interleukin-6 (IL-6) inhibition, in combination with immunosuppressive therapy with variable success.

Here we report a case of an American male with biopsy proven EBV positive TAFRO syndrome, who was successfully treated with therapies focused on cytokine reduction (tocilizumab), immunosuppression (rituximab), and cytotoxicity (etoposide). His case may help elucidate a possible viral etiology for the disease and also demonstrates a novel, successful treatment regimen that dampened the hypercytokinemia response which is uniformly seen in TAFRO patients and ultimately normalized this patient's symptoms and organ dysfunction.

## 2. Case Presentation

### 2.1. Clinical Presentation

**Figure 1:**
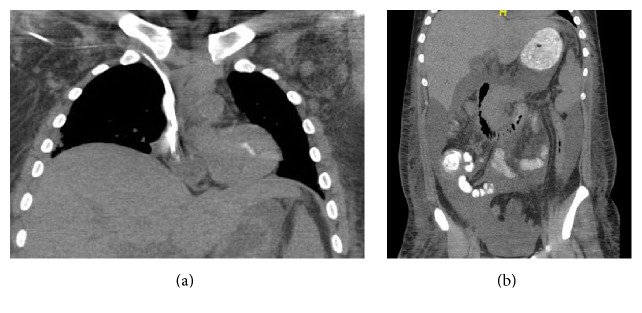
Computed tomography demonstrating diffuse axillary lymphadenopathy measuring 1 to 1.2 cm in short axis (a) and ascites (b).

A 22-year-old American male presented to an outside hospital with an initial complaint of pleuritic chest pain, shortness of breath, and lightheadedness. Prior to the admission, he had two weeks of fevers, night sweats, and malaise and one week of constipation, nausea, and abdominal discomfort. Vital signs were significant for fever, tachycardia, tachypnea, and hypoxia. Physical exam was notable for a diaphoretic, well-nourished male with bilateral lower lung crackles, hepatosplenomegaly, and bilateral axillary lymphadenopathy. Laboratory studies were remarkable for acute renal failure (creatinine 2.28 mg/dL, reference range 0.6–1.3 mg/dL, leukocytosis (WBC) 37 × 10^9^/L, and reference range: 3.5–11 × 10^9^/L). Computed tomography demonstrated diffuse axillary lymphadenopathy (1 to 1.2 cm in diameter), hepatomegaly, and anasarca (Figures 1(a) and 1(b)). He was then transferred to Rhode Island Hospital, where he progressed to severe thrombocytopenia (platelets count 2 × 10^9^/L), anemia (hemoglobin 7.7 g/dL), anuric renal failure with hyperuricemia, hyperkalemia, hypocalcemia, and hyperphosphatemia requiring emergent dialysis, and hypoxic respiratory failure necessitating intubation.

### 2.2. Diagnostic Course

**Figure 2:**
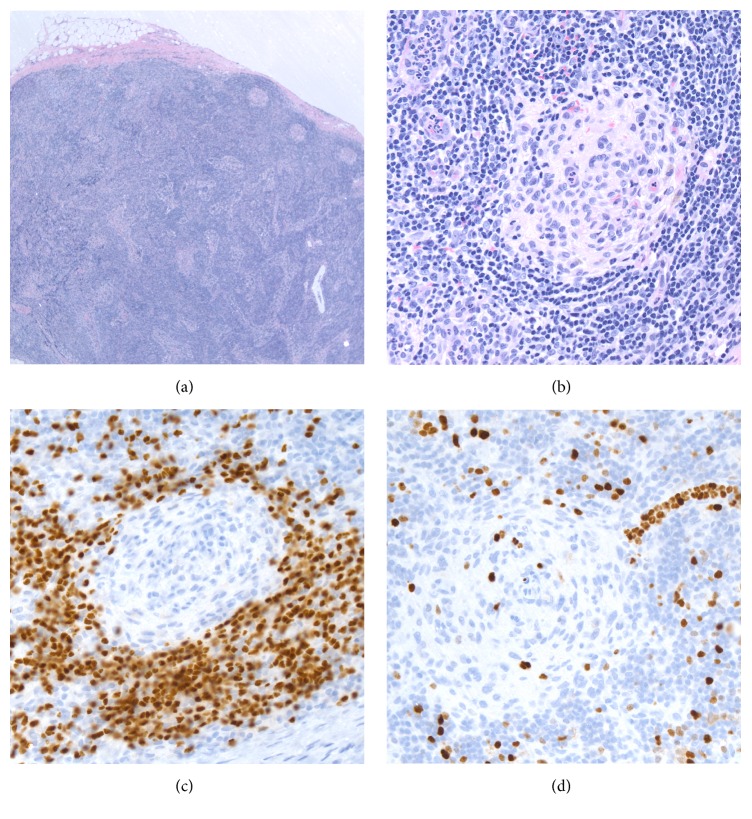
Biopsy of left axillary lymph node demonstrating (a) well-developed germinal centers with round to ovoid to slightly irregular contours, (b) small atrophic germinal centers, (c) depleted of PAX-5 positive B lymphoid cells with very low proliferation (d) rates using MIB-1 antibody.

**Figure 3:**
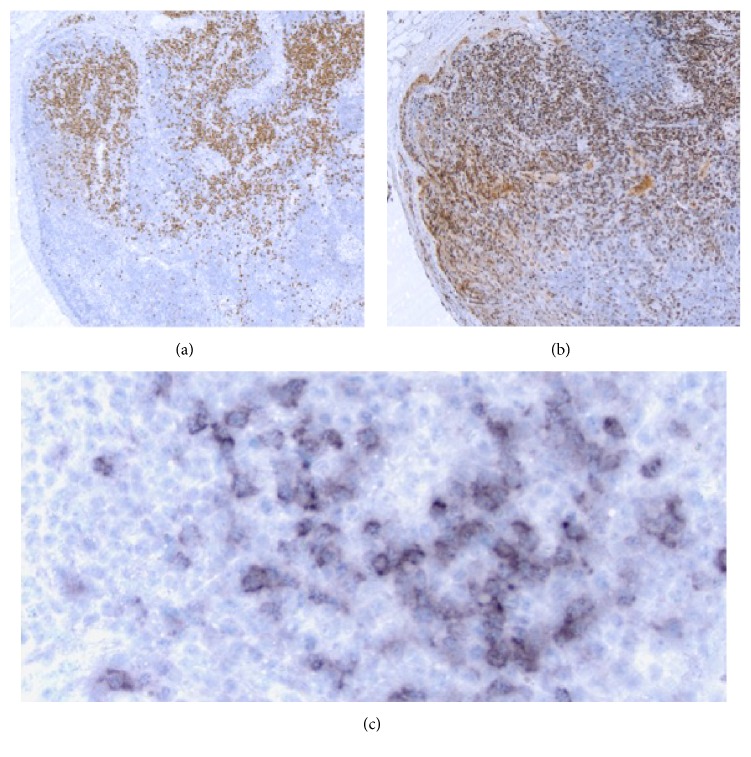
Biopsy of left axillary lymph node, with increased numbers of MUM1 positive plasma cells in the interfollicular areas ((a) objective 50x) and with a high proliferation rate ((b) MIB-1 antibody, objective 500x). EBER in situ hybridization stain with increased subset of positive cells in the interfollicular area ((c) objective 500x).

**Figure 4:**
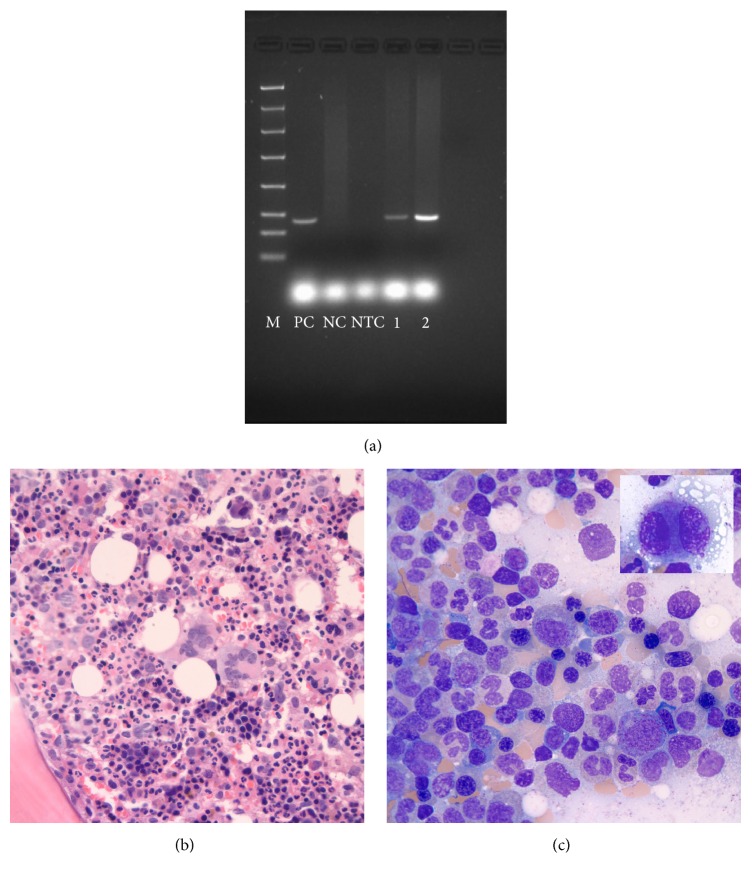
PCR gel for detection of EBV DNA: samples 1 and 2 are two replicates of patient's DNA with different amounts of sample; M is the 100 bp ladder molecular weight marker. PC, positive control; NC, negative control; NTC, no template control (a). Hypercellular bone marrow biopsy ((b) hematoxylin and eosin stain 50x, immersion oil) and aspirate ((c) Wright Giemsa stain, 100x, immersion oil) with trilineage hematopoiesis; a small hypolobated megakaryocyte ((c) inset).

Biopsy of the left axillary lymph nodes was remarkable for scattered secondary lymphoid follicles variable in size, some with well-developed germinal centers with round to ovoid to slightly irregular contours to a few secondary lymphoid follicles with small atrophic germinal centers (Figures 2(a) and 2(b)) depleted of B lymphoid cells (Figure 2(c), PAX-5 antibody) and with very low proliferation rates (Figure 2(d), MIB-1 antibody). There was interfollicular polytypic plasmacytosis, composed mostly of mature-appearing MUM1 positive plasma cells (Figure 3(a)) associated with a high proliferation rate (Figure 3(b), MIB-1 antibody), and there was a subset of lymphoid cells in the interfollicular region with predominant cytoplasmic positivity for EBER in situ hybridization (Figure 3(c)). By flow cytometry immunophenotypic analysis identified a larger CD3 positive T-cell population (77% of the lymphoid cells analyzed) in the lymph node sample examined with a CD4-to-CD8 cell ratio of 4.3 : 1 with partial loss of CD7 in a small subset of the T-cells analyzed. The smaller (21% of the lymphoid cells analyzed) CD19 positive B lymphoid population was polytypic with a surface immunoglobulin kappa to surface immunoglobulin lambda of 1.3 and 1% NK cells were also identified. Polymerase chain reaction (PCR) studies did not detect immunoglobulin heavy chain gene rearrangements or T-cell receptor beta and gamma gene rearrangements but detected the presence of EBV DNA sequences (Figure 4(a)). The above findings are in keeping with an atypical lymphoid hyperplasia with features of a mixed type of Castleman disease.

Patient's bone marrow examination was remarkable for hypercellularity with trilineage hematopoiesis (Figure 4(b)) with mild dyserythropoiesis (Figure 4(c)) and dysmegakaryopoiesis (Figure 4(c) inset) but was associated with reported normal 46, XY karyotype. There was mild increase in reticulin deposition in patient's bone marrow. Of interest, flow cytometry analysis of the peripheral blood identified a drastic reduction of the CD4 positive T-cell subset with absolute numbers of CD8 positive T-cells, B-cells, and NK cells within the normal reference ranges. The CD4-to-CD8 cell ratio was reversed (0.37 : 1). Flow cytometry analysis of his ascitic fluid identified a predominant CD3+ T-cell population (90% of the analyzed lymphoid cells) with a reversed CD4-to-CD8 cell ratio (0.25 : 1).

Overall, based on the constellation of clinical and pathological data the patient was diagnosed with TAFRO syndrome. Additional work-up for TAFRO syndrome included an elevated IL-2R of 8944 (reference range 622–1619 pg/mL), IL-6 596 (reference range 0.31–5 pg/mL), and CRP 30 (reference range 0–10 mg/ml).

### 2.3. Treatment Course and Outcome

Upon the diagnosis of TAFRO syndrome, the patient was initially started on sole anti-IL-6 therapy with tocilizumab, 8 mg/kg every 2 weeks. Due to worsening clinical status he was then treated with weekly rituximab 375 mg/m^2^ and etoposide 100 mg/m^2^ (dose reduced 50% due to renal failure). Following this regimen, the patient showed drastic clinical improvement in symptoms, laboratory findings, and imaging. He was followed up as an outpatient where he completed an eight-week, weekly course of tocilizumab, concomitantly with a four-week course of weekly etoposide and rituximab. By the eighth week, his anasarca, organomegaly, and lymphadenopathy resolved and his renal function normalized. In addition, his complete blood count normalized, IL-6 decreased to 123 pg/mL, and CRP normalized to less than 20 mg/ml. He remains symptom-free from the disease after almost 2 years from the diagnosis.

## 3. Discussion

TAFRO syndrome, an atypical variant of Castleman disease, is a rare constellation of symptoms that has only recently been described.

### 3.1. Viral Etiology for TAFRO Syndrome

The patient described in this case report was positive for EBV DNA sequences by PCR in both the biopsied lymph nodes and bone marrow sample and had a subset of positive lymphoid cells by EBER on in situ hybridization. To date, no known virus has been associated with TAFRO syndrome; however, certain viruses are associated with MCD, namely, HHV-8 in HIV positive patients and EBV. This is a novel finding and may be a contributor to the etiology of TAFRO syndrome. In addition, the lymph node findings with a marked discrepancy between the low proliferation rate noted in atrophic germinal centers depleted of B lymphoid cells and the morphologically mature-appearing plasma cell proliferation noted in the interfollicular regions, associated with a high proliferation rate, may underline an extreme immune response leading to exhaustion of the germinal center cells and differentiation toward plasma cells secondary to strong antigenic stimulation. Furthermore, the reversed CD4-to-CD8 T-cell ratio identified in the peripheral blood and ascitic fluid in our patient, which initially raised in the differential diagnosis a possible HIV infection, has been noted in patients with other acute viral infections such as EBV and CMV, supporting also the possibility of an EBV driven TAFRO syndrome [6].

### 3.2. Treatment

Due to the rarity of TAFRO syndrome there are no guidelines to treatment. Also, the ambiguity of presenting symptoms and findings complicates treatment directions. In review of previous TAFRO cases, using PubMed search terms for confirmed TAFRO diagnosis, patterns of treatment can be seen (Table 1). Most cases involved treatment with steroids [7–13]. Unfortunately, symptomatic improvement gained from steroids alone has usually been reported as short-lived [2, 14].

In several cases, immunotherapeutic agents were utilized. Rituximab, a monoclonal antibody directed against the CD20 antigen on B-lymphocytes, has shown durable responses in HIV positive MCD [14]. Two cases of TAFRO syndrome reported clinical improvement with rituximab [8, 10]. In one case, it was used as monotherapy and, in the other, rituximab was used in combination with methylprednisolone and tocilizumab. Cyclosporin A was documented to have resulted in complete remission in four cases [8, 13].

Ideally chemotherapy would provide a means to eliminate large portions of hypercytokine-secreting cells; however, its use without immunosuppressive therapy has not had lasting and meaningful results [12, 15]. Only one patient in the literature [16] had been treated with regimen consistent of rituximab, tocilizumab, cyclophosphamide, and etoposide.

Since elevations in IL-6 have been thought to contribute to the pathophysiology of multicentric Castleman disease (MCD), targeting this pathway has been one of the primary focuses of treatment. Tocilizumab (TCZ) is a monoclonal antibody targeting the IL-6 receptor. In the three cases documenting its use in TAFRO syndrome, TCZ was combined with steroids resulting in improvement of symptoms and decreased markers of inflammation [7, 9, 11].

As with the other TAFRO cases, our patient's course was rapidly progressive. However, the combination of steroids, etoposide, rituximab, and TCZ was very effective in arresting the disease and reversing its end organ damage. This combination of therapy may be a novel effective approach in the management of this disorder and warrants consideration for this rare disease in future patients, especially if new guidelines are being investigated to treat TAFRO based upon disease severity [5].

## 4. Conclusion

TAFRO syndrome can be a rare, aggressive life-threatening disorder associated with multicentric Castleman disease which has only recently been described. The cases reported thus far have been almost exclusively in the Japanese literature. We present a case of a young American male to add to the body of literature of this unusual disorder and propose a possible viral cause and successful treatment regimen for those who have evidence of multiorgan failure.

## Figures and Tables

**Table 1:** Summary of the most common treatment regimens for those diagnosed with TAFRO syndrome.

Case report	Age (years)/gender	TAFRO-directed treatment			General outcome
		1st line	2nd line	3rd line	
Simons, 2016	22/M	TCZ	TCZ, rituximab, etoposide		Clinical improvement

Tedesco, 2015	21/F	Prednisone, TCZ	Rituximab, cyclophosphamide, vincristine, prednisone		Clinical improvement

Takai et al., 2010 [17]	39/M	Methylprednisolone, prednisolone			Clinical improvement
	38/M	Prednisolone			Clinical improvement

Koduri et al., 2014 [15]	14/M	CHOP × 6	Thalidomide		Persistent lymphadenopathy, no edema or ascites

Kubokawa et al., 2014 [7]	15/M	Methylprednisolone	TCZ, IVIG		Cytokine levels decreased by 4 months

Ozawa et al., 2014 [10]	48/F	IVIG	Steroids	Rituximab	Resolution of thrombocytopenia and marrow fibrosis

Kawabata et al., 2013 [9]	47/F	Prednisone	TCZ, steroids		Improvement of IL-6 levels, no signs of disease recurrence

Inoue et al., 2013 [8]	49/F	Dexamethasone	Cyclosporine A		Resolution of thrombocytopenia and marrow fibrosis

Iwaki et al., 2013 [11]	43/F	Methylprednisolone	Rituximab, TCZ, steroids		Improvement of thrombocytopenia and anasarca

Masaki et al., 2013 [12]	57/F	Methylprednisolone	CHOEP		Died from septic shock
	73/M	Prednisolone			Died from multiorgan failure

Takai et al., 2010 [17]	47/F	CHOP, prednisolone	Prednisolone (maintenance)		Clinical improvement
	56/M	Methylprednisolone, IVIG	Cyclosporin A		Improved platelet count, edema, ascites
	49/M	Methylprednisolone, IVIG			Died from multiorgan failure
	53/F	Prednisolone	Prednisolone, cyclophosphamide		Clinical improvement
	56/F	Methylprednisolone, cyclosporin A			Clinical improvement

Abdo et al., 2014 [18]	81/M	Prednisone			Clinical improvement

## References

[1] Kawabata H., Takai K., Kojima M. (2013). Castleman-Kojima disease (TAFRO syndrome) : a novel systemic inflammatory disease characterized by a constellation of symptoms, namely, thrombocytopenia, ascites (anasarca), microcytic anemia, myelofibrosis, renal dysfunction, and organomegaly : a status report and summary of Fukushima (6 June, 2012) and Nagoya meetings (22 September, 2012). *Journal of Clinical and Experimental Hematopathology*.

[2] Fajgenbaum D. C., van Rhee F., Nabel C. S. (2014). HHV-8-negative, idiopathic multicentric Castleman disease: novel insights into biology, pathogenesis, and therapy. *Blood*.

[3] Chen C.-H., Liu H.-C., Hung T.-T., Liu T.-P. (2009). Possible roles of Epstein-Barr virus in Castleman disease. *Journal of Cardiothoracic Surgery*.

[4] Barozzi P., Luppi M., Masini L. (1996). Lymphotropic herpes virus (EBV, HHV-63 HHV-8) DNA sequences in HIV negative Castleman's disease. *Journal of Clinical Pathology—Clinical Molecular Pathology*.

[5] Masaki Y., Kawabata H., Takai K. (2016). Proposed diagnostic criteria, disease severity classification and treatment strategy for TAFRO syndrome, 2015 version. *International Journal of Hematology*.

[6] Wakiguchi H., Hisakawa H., Kubota H., Kurashige T. (1999). Strong response of T cells in infants with dual infection by Epstein-Barr virus and cytomegalovirus. *Pediatrics International*.

[7] Kubokawa I., Yachie A., Hayakawa A. (2014). The first report of adolescent TAFRO syndrome, a unique clinicopathologic variant of multicentric Castleman's disease. *BMC Pediatrics*.

[8] Inoue M., Ankou M., Hua J., Iwaki Y., Hagihara M. (2013). Complete resolution of TAFRO syndrome (thrombocytopenia, anasarca, fever, reticulin fibrosis and organomegaly) after immunosuppressive therapies using corticosteroids and cyclosporin A: a case report. *Journal of Clinical and Experimental Hematopathology*.

[9] Kawabata H., Kotani S.-I., Matsumura Y. (2013). Successful treatment of a patient with multicentric castleman's disease who presented with thrombocytopenia, ascites, renal failure and myelofibrosis using tocilizumab, an anti-interleukin-6 receptor antibody. *Internal Medicine*.

[10] Ozawa T., Kosugi S., Kito M. (2014). Efficacy of rituximab for TAFRO syndrome, a variant type of multicentric Castleman's disease. *Rinshō Ketsueki*.

[11] Iwaki N., Sato Y., Takata K. (2013). Atypical hyaline vascular-type castleman's disease with thrombocytopenia, anasarca, fever, and systemic lymphadenopathy. *Journal of Clinical and Experimental Hematopathology*.

[12] Masaki Y., Nakajima A., Iwao H. (2013). Japanese variant of multicentric castleman's disease associated with serositis and thrombocytopenia—a report of two cases: is TAFRO syndrome (Castleman- Kojima disease) a distinct clinicopathological entity?. *Journal of Clinical and Experimental Hematopathology*.

[13] Takai K., Nikkuni K., Momoi A., Nagai K., Igarashi N., Saeki T. (2013). Thrombocytopenia with reticulin fibrosis accompanied by fever, anasarca and hepatosplenomegaly : a clinical report of five cases. *Journal of Clinical and Experimental Hematopathology*.

[14] El-Osta H. E., Kurzrock R. (2011). Castleman's disease: from basic mechanisms to molecular therapeutics. *Oncologist*.

[15] Koduri P. R., Parvez M., Kaza S., Pappu P., Anuradha S. (2014). Castleman-Kojima disease in a South Asian adolescent. *Journal of Clinical and Experimental Hematopathology*.

[16] Iwaki N., Fajgenbaum D. C., Nabel C. S. (2016). Clinicopathologic analysis of TAFRO syndrome demonstrates a distinct subtype of HHV-8-negative multicentric Castleman disease. *American Journal of Hematology*.

[17] Takai K., Nikkuni K., Shibuya H., Hashidate H. (2010). Thrombocytopenia with mild bone marrow fibrosis accompanied by fever, pleural effusion, ascites and hepatosplenomegaly. *Rinsho Ketsueki*.

[18] Abdo L. A., Morin C. P., Collarino R. P., Cabane J. P., Gatfosse M. A. (2014). First European case of TAFRO syndrome associated with Sjogren disease. *American Journal of Internal Medicine*.

